# Unraveling the South Asian enigma: concurrent manifestations of child anthropometric failures and their determinants in selected South Asian countries

**DOI:** 10.1186/s40795-023-00771-4

**Published:** 2023-10-30

**Authors:** Sabeen Saif, Sofia Anwar

**Affiliations:** Department of Economics, Government College University, Faisalabad, Pakistan

**Keywords:** CIAF, Air pollution, Mothers’ education, Malnutrition, Cointegration, South Asia, anthropometric failure

## Abstract

Malnutrition among children is pervasive in South Asia and there are also reports of overnutrition. To better understand this phenomenon, we need a composite measure. However, the existing measures such as CIAF (Composite Index of Anthropometric Failure) and its revised version have ignored the overnutrition aspect of the phenomenon. This study proposes an extended version of CIAF which also considers overnutrition. This new measure was compared with the existing measures by using data from 1990 to 2018 for three selected South Asian countries including Pakistan, India and Bangladesh. We also examined the effects of socioeconomic and environmental variables on the outcome variable. The results reveal that the new measure (ECIAF) is better at measuring the phenomena. The burden of overall malnutrition has been decreased in the region. However, an increase in the concomitant prevalence of wasting and underweight is observed in both Pakistan and India and stunting and overweight is observed only in India. Besides, political stability, prevalence of undernourishment, anemia in children, mother’s education, household size, dependency ratio, air pollution and unimproved sanitation are significantly correlated with childhood malnutrition. The findings also testified to long-run cointegrating relationship among the variables.

## Introduction

Health is an engine of economic growth [1]. The health of women, mothers and children in particular is fundamental to economic development [2]. Healthy children are the linchpin for healthy and thriving societies as reflected by the agenda of Millennium and Sustainable Development Goals [3]. Child health affects economic growth directly and indirectly in many ways. Directly it plays a pivotal role in establishing the foundations of human capital investment [4] and reduction in the economic burden of illnesses [5]. Indirectly it impacts economic growth by affecting the future income of people through the impact, health has on education such as schooling and cognitive skills [6, 7].

Child malnutrition manifests in three broad forms: undernutrition, which includes stunting (height-for-age), wasting (weight-for-height), and underweight (weight-for-age), overnutrition (overweight and obesity) and micronutrient-related malnutrition [8]. There is no such confirmatory test to measure undernutrition and overnutrition. Anthropometry is a pragmatic and immediately applicable technique for measuring children’s development patterns during the first five years of his/her life. It quantifies the nutritional situation at one point in time and allows comparisons over time [9].

### How serious is malnutrition?

Child nutritional anthropometry is the most pressing problem in the world, damaging to both children and nations. According to the WHO global estimates (2021), around 149.2 million children under 5 years of age suffered from stunting, 45.4 million were affected by wasting, 20.5 million were underweight and 38.9 million were too heavy for their height. These statistics are staggering and confirm that malnutrition is a global health issue. Moreover around 89% of those stunted, 93% of wasted and 77% of overweight children are more likely to live in low and middle- income countries. Most of them are the sub-regions of Africa and Asia [10]. Although some efforts are being made towards reducing malnutrition and as a result, stunting is declining steadily in all regions of East, Pacific, and South Asia except Africa [11]. However, still, only one-quarter of all countries are ‘on track’ to halve the number of children affected by stunting by 2030. On the other hand, wasting persists at alarming rates and it seems quite impossible to achieve the 2030 wasting targets for nearly half of countries [12]. Whereas, overweight prevalence is increasing in the majority of the countries, analysed in Southern Africa, Oceania, South-eastern Asia, South America and the Caribbean and therefore requires a reversal in trajectory to meet the required targets [10].

More than half of the malnourished children of South Asia live mainly in Pakistan, Bangladesh and India. This is the main reason that this study focused on these three countries for analysis. Children in the region, have some highest global rates of stunting and wasting, also known as South Asian enigma [13]. The number of stunted children under the age of five is around 38% in Pakistan, 32% in Bangladesh and 30% in India. According to the global nutrition report, Pakistan and India was reported the home of almost half of all stunted children around the globe carrying 10.7 million and 46.6 million stunted children respectively [14]. India is home to the world’s most wasted children (25.5 million). Bangladesh is ‘on course’ to prevent childhood wasting but still, 9.8% children are affected by wasting which is higher than the average for the Asia region (8.9%). Pakistan is also making some progress towards achieving the target for wasting but still, 7.1% children are affected. The report also highlights the worrying incidence and universality of malnutrition in all its forms the changing face of malnutrition in Asia. While, the prevalence of overweight among children under five is 2.5%, 1.6% and 2.4% in Pakistan, India and Bangladesh respectively [15]. All this indicates that despite some achievements and partial success, the current pace of change is too slow to achieve the nutrition targets by 2030 in the majority of countries.

Moreover, South Asia has seen a large increase in the “double burden” of malnutrition in recent years. At the individual level, it was observed by the concurrent manifestations of multiple anthropometric failures, undernutrition represented by wasting, stunting, or underweight co-occurring with overnutrition overweight/obesity or subsequent overweight in a malnourished child under five. This complex phenomenon constitutes an unprecedented challenge to global public health and has been prioritized by international health organizations, prompting governments to accurately measure the coexisting forms of malnutrition and take swift action [16].

## Research objective

One way to achieve those nutrition targets is to accurately measure the overall burden of malnutrition among children under five (outcome variable) and find the factors that are responsible for this exceptional scale of undernutrition and overnutrition. Empirically, many studies have tried to fulfill the underlying objective, see, e.g. [17–27]. All of these studies have tried to determine child malnutrition status in South Asia by using conventional indices of anthropometric failures. Though these standard estimates mirror distinct biological processes but have the problem of overlapping. Thus they are unable to provide the correct and comprehensive measure of undernourishment among the study subjects. For instance, a number of stunted children will also have wasting; others might be experiencing the concurrent burden of stunting and underweight. Some children might face a concomitant prevalence of all three indicators. Consequently, these conventional indices can’t truly detect the overall burden of malnutrition [28]. To fill this gap some studies have used CIAF as proposed by Peter Svedberg [29] and its revised version by Nandy [30]. These include [31–34]. Firstly all those studies only focus on and estimate undernutrition and overlook overnutrition. In this way, did not address the issue of a concomitant coexistence of stunting with overweight among children under five. Over the last three decades, the high prevalence of overweight among children has been confirmed by many studies [35, 36]. Several recent studies have reported the presence of overweight and obesity simultaneously with stunting, i.e., low HAZ among children under five [37, 38]. Secondly, none of those studies have been done exclusively on South Asia using the CIAF model to identify the hidden vulnerabilities among children under five. In this context, the extended model of CIAF, employed by this study represents a better estimate of the overall burden of malnutrition among children as it measures both undernutrition and overnutrition simultaneously. Accordingly, this study would fill this gap by estimating the extent and pattern of malnutrition among children and by exploring further dimensions of the hidden malnutrition “iceberg”.

The second aim is to examine, what are the most proximate factors, especially environmental indicators that are responsible for this concomitant prevalence of anthropometric failures among children to contribute to the existing body of evidence needed for the formulation of effective interventions. Are they the same as in case of individual conventional indices? Besides, there is a dearth of evidence on potential confounders of malnutrition in children under five focusing in specific on socioeconomic and environmental factors utilizing the latest 2018 PDHS data set. Perceptibly, using a panel modeling allows us to see the impact of those indicators not reported in DHS. Moreover this study measures the interaction of sanitation and ambient air pollution on child malnutrition. Although literature is available on sanitation facilities and air pollution in Pakistan yet no study analyze the dual effect of sanitation and air pollution in the case of Pakistan. These are the gaps that this study tried to cover.

## Predictors

Variables used to decompose the change in the coincidence of growth disorders among children under five were demographic and socioeconomic characteristics of household (household size, dependency ratio, female literacy rate and political stability), nutritional characteristics (prevalence of anemia among children under five and prevalence of undernourishment), and environmental characteristics (unimproved sanitation facilities and ambient particulate matter pollution). These variables are correlated with child nutritional status in prior empirical studies. The theoretical justification of the variables used in the multivariate analysis is given below turn by turn.

### Female literacy rate

Social determinants of child health such as female education are strongly associated to health-seeking behavior and improving overall health outcomes of their children. Education of women reflected as higher literacy rates are related to higher incomes and better health indicators such as lower infant mortality, child malnutrition and population growth rates. Women’s education has a ‘multiplier effect’ on the well-being of their children [39]. Educated women tend to marry at a later age, have fewer children and be more informed about nutrition requirements and healthcare practices of their children [40]. Therefore there exists a negative correlation between female literacy rate and child death [41]. Thus, the coefficient α is expected to be negative.

### Ecological quality indicators

This study used two indicators of ecological quality; one is air pollution and the other is unimproved sanitation facility. Emerging evidence suggests that adverse ecological conditions and pollution are major contributors to childhood malnutrition and mortality, particularly in developing countries. Children are particularly vulnerable to certain environmental risks, including air pollution and inadequate sanitation [42]. Another study also found a potential relationship between ambient air pollution and child growth indicators [43]. Similarly, Unimproved sanitation was also found to be a significant predictor of anthropometric failures among children under five [44–46]. Khan et al. (2021) assessed that sanitation in terms of the sanitation ladder frequently contributes to child growth failures and may bring the source of fecal contamination to the doorstep of the households [47]. Therefore environmental risks have an impact on the health and development of children [42]. Thus, the coefficient α is expected to be positive.

### Political stability

Malnutrition is often considered a political problem as the constant instability aggravates the food and sanitation situation in the country [48]. Political stability rests on a government’s ability to carry out its proclaimed programs and provide reliable public services to the commons. It creates conditions that are conducive to the economic stability of the households, the functioning of markets for essential nutrition inputs such as food and keep food price at levels [23]. Children are particularly vulnerable to food insecurity resulting from food price spikes. The effects are likely to be an increased incidence of stunting, wasting and other growth disorders among children [49]. Therefore it is claimed that political stability is estimated to have large and permanent effects on nutrition status and plays a significant role in reducing childhood undernutrition along with other socio-demographic factors [50]. Thus, the coefficient α is expected to take a positive sign.

### Prevalence of undernourishment

Undernourishment refers to the condition of insufficient intake of food. It can lead to serious health issues, including impaired growth and obesity in children [51, 52]. Therefore the effect of complementary feeding practices is reflected in the severely jeopardized health of children under five. The coefficient of Prevalence of undernourishment is contemplated to be positive.

### Anemia in children

Prieto-Patron et al. (2018) identified that severe chronic anemia may lead to child health variables such as stunting, wasting, underweight and overweight [53]. The likely cause of childhood anemia is delayed growth problems among children under five [54]. Thus, the coefficient is expected to take a positive sign.

### Dependency ratio

Malnutrition is not only a health sector problem. Demographic factors like high household dependency ratios [55] and large household size [56], which are mainly the social determinants, are detrimental to children’s nutritional outcomes and inequalities. Thus, the coefficient is expected to take a negative sign.

### Household size

As household size increases malnutrition status wasting, stunting, overweight and underweight increases, because with family size increase, resources become scarce and less nutrition and care focused on children. Thus, the coefficient is expected to take a negative sign.

The study also attempts to examine the impact of economic growth and health expenditures as a percentage of GDP on child undernutrition in South Asia but finds no synergistic effects and the value of the coefficient of economic growth and public health expenditure will remain insignificant in the equation of child’s malnutrition status. The role of economic growth in reducing child undernutrition remains an open and highly debated question. Economic growth does not necessarily help countries to decrease undernourishment [57]; this outcome is only found significant for South Asia. Economic growth is indispensable but not enough factors require combating undernourishment. The reason could be the substantial disparity in the share of poor people in the aggregate economic growth [18]. Similarly, the actual relationship between health spending and child health is still unclear, particularly at the macro level as most of the researchers found an insignificant association between health expenditure and under-five malnutrition. Healthcare expenditures as a percentage of GDP are not a dominant driver of childhood malnutrition [58]. Household water supply facilities were also not significantly associated with the concurrent prevalence of children’s anthropometric failures.

## Data and methodology

### Data sources and description

Nationwide cross-sectionals have been employed to capture the health status of children under five over the past three decades in Pakistan, India and Bangladesh. There are five available cross-sections for Pakistan from 1990 to 2018. These include PDHS (1990–1991), NNS (2001–2002), NNS (2011), PDHS (2012–2013) and PDHS (2017–2018). PDHS (2006–2007) is not utilized for the current analysis as it doesn’t consist of anthropometric indicators. For this reason, the study used NNS (2001) to fill the huge time gap between 1991 and 2012. Similarly Indian available data sets for the same time period are IDHS (1992-93), (1998–1999), (2005–2006) and (2015–2016). Seven data sets are available for Bangladesh. These include BDHS (1990), (1996–1997), (2004), (2007), (2011), (2014) and (2017–2018).

Data for exposure variables such as female literacy rate, household size, dependency ratio, the prevalence of Anemia in children and prevalence of undernourishment is taken from (WDI) and World Bank data indicators for Pakistan, Bangladesh and India for the period of 1990 to 2018. Data for political stability was derived from (ICRG) as one of the six dimensions of good governance. Data for household unimproved sanitation facilities was extracted from the WASH data source. Data for ambient particulate matter pollution was downloaded from GBD data visualizations and the Global Health Data Exchange (GHDx), IHME’s catalog of the world’s health and demographic data.

### Methodology

The methodological approach consists of zooming in on this data through several interrelated steps. The first step involves the construction of an extended version of a composite index of anthropometric failures (ECIAF) which represents our main contribution to a better operationalization of malnutrition among children under five within the selected region. We identified the vulnerable distinct subgroup as pointed out in Table 1 and compared the ECIAF index with other conventional indices. The next step incorporates the shifting conditions of undernutrition and overnutrition concerning different periods within the selected region. The third step involves different econometrics steps to investigate the association between child anthropometric failures and socioeconomic and environmental determinants. Firstly we check the data stationarity through the unit root test. After confirming that the data is stationary at first difference, we will check the cointegration. This test is used to find the long- run association between the dependent variable and explanatory variables. For this purpose, Kao and Johansen’s - Fisher Panel cointegration tests have been employed. These tests are very effective and suitable for Panel study. The last step is to employ the advanced panel data econometric techniques known as fully modified ordinary least squares (FMOLS) along with descriptive statistics. The FMOLS model is best to employ because it directly evaluates the long-run impact of the independent variables on the dependent variable after correcting for the endogeneity bias and small sample bias in the time series by taking the leads and lags of the first-differenced regressors. FMOLS estimator also takes into account the nuisance parameters and possible autocorrelation and heteroscedasticity phenomena of the residues.

The study would examine the short-run affiliation and long-run association between nominated potential confounders and child anthropometric failures for selected South Asian countries (Pakistan, Bangladesh and India).

### Panel econometric equation

ECIAF f (Dependency ratio, Household size, Female literacy rate, Prevalence of undernourishment, Prevalence of anemia in children under five, Air pollution, unimproved sanitation facilities and political stability).

ECIAF = *β*^0^ + *β*^1^ (Dependency ratio) +*β*^2^ (Household size) + *β*^3^ (Female literacy rate) + *β*^4^ (Prevalence of undernourishment) + *β*^5^ (Prevalence of anemia) + *β*^6^ (Air pollution) + *β*^7^ (Unimproved sanitation facilities) + *β*^8^ (Political stability) + *µ* it *E*CIAF *= β*^0^ + *β*^1^ (DR) +*β*^2^ (HHS) + *β*^3^ (FLR) + *β*^4^ (POU) + *β*^5^ (POA) + *β*^6^ (AP) + *β*^7^ (USF) + *β*^8^ (PS) + *µ* it.

This study has calculated WHZ, WAZ and HAZ scores by using ENA for smart software concerning WHO standards. Descriptive analysis and other computations have been done utilizing SPSS version 20. Panel econometric analysis has been done by using EViews 12.

### Operationalization of ECIAF, the outcome variable

The first step involves estimating the anthropometric indices. Anthropometric indices are constructed using the information on children’s weight, recumbent length, (< 24 months or child unable to stand without support) stature (> 24 months), age in months, and gender. Four key anthropometric indicators are calculated, these include height for age (for stunting), weight for age (for underweight), weight for height (for wasting) and overweight. Stunting refers to impaired growth and development, experienced by children less than five years of age, used as the marker of chronic malnutrition. In more logical terms, stunting can be defined as height for age z scores (<-2 SD), below the average according to the WHO child growth standards. Wasting signifies a severe course of weight loss and is defined as weight for age z scores (<-2 SD), below the average according to the WHO child growth standards. Underweight for age mirrors body mass corresponding to chronological age and a diagnostic of weight for age z scores (<-2 SD) concerning the WHO standards. Whereas overweight can be examined as a combination of two terms, high weight for height and high weight for age. It is defined as weight for height z scores and weight for age z scores (> 2 SD) above the average according to the WHO reference values [59, 60].

There are three generally accepted procedures for assessing child growth statuses. Among these, the study used the method of creating z scores. In the first step, we took the difference between the child’s height or weight (relative to the age and gender) and the mean/median values for the reference population. Then in the second step Z score is computed by dividing this difference by the standard deviation of the reference group. This can be written as follows in the case of calculating height for age z scores.

1 $$Z-score=\frac{\text{H}\text{i} - \text{H}\text{r}}{Standard deviation of the refrence population}$$

Where, Hi stands for the estimated height of the child and Hr is the median height of the reference group.

The number of children whose z score is below minus 2SD is undernourished. The World Health Organization proposed a reference population. This reference is formed on the basis of the anthropometric indicators of the children of six countries [61, 62].

### ECIAF model

The ECIAF model with mutually exclusive categories is elaborated under.

**Table 1:** A portrayal of the categories of ECIAF among children

Groups	Descriptions	Explanation of the levels	Wasting	Stunting	Under weight	Over weight
**A**	No failure	Standard levels of WAZ,WHZ & HAZ	No	No	No	No
**B**	Wasting only	Only WHZ is below − 2 SD	Yes	No	No	No
**C**	Wasting & Underweight	WHZ & WAZ are below − 2 SD, but HAZ is in a normal range	Yes	No	Yes	No
**D**	Wasting, Stunting & Underweight	WHZ, HAZ & WAZ, all are below − 2 SD	Yes	Yes	Yes	No
**E**	Stunting & Underweight	HAZ & WAZ, are below − 2 SD, but WHZ is in a normal range	No	Yes	Yes	No
**F**	Stunting only	Only HAZ is below − 2 SD	No	Yes	No	No
**G**	Stunting &Overweight	If WHZ & WAZ are above 2 SD	No	No	No	Yes
**H**	Overweight only	If HAZ is below − 2 SD and WHZ & WAZ are above 2 SD	No	Yes	No	Yes
**Y**	Underweight only	WAZ is below − 2 SD	No	No	Yes	No

Table 1 counts all children with wasting and/or stunting and/ or overweight and/ or underweight sub-grouped in eight different categories (Groups B-H and Y) and excludes the first category (group A), children with no anthropometric failure. Groups B, F, H and Y consisted of children vulnerable to only one kind of growth retardation problems while groups C, D, E and G were composed of children with concurrent manifestations of anthropometric failures.

### ECIAF model equation

Lastly, the following formula has been proposed to detect normal, undernourished and overnourished children among the studied populations.

2 $$ECIAF=\frac{1-A}{\left((A+B+C+D+E+F+Y\right)+ (G+H))}$$

**(Source: Kuiti & Bose, 2018)** [63].

### Definition and construction of predicted variables

The dependency ratio, household size, mother’s education, the prevalence of undernourishment within the population, anemia in children, household unimproved sanitation, air pollution and government stability are the selected as the potential risk factors. The dependency ratio is calculated by adding the percentage of children under the age of 15 years and older population above the age of 64 years divided by the percentage of independents in the household (15–64) year then multiplied by 100. Household size is the total number of members in a family. Mother’s education is the total number of years of schooling of a child’s mother. Prevalence of undernourishments is the percentage of the population whose habitual food intake is not enough to provide the dietary energy levels, required to maintain a normal active and healthy life. Child anemia is a condition referred to as low hemoglobin lack of enough healthy red blood cells, or high rates of red blood cell destruction among children under five. Environmental degradation is proxied by air pollution and household unimproved sanitation. Air pollution is the ambient particulate matter pollution (micrograms per cubic meter) and unimproved sanitation is the share of the population with access to unimproved sanitation facilities.

## Child malnutrition trends within the selected region

An amalgam of anthropometric failures for children has been calculated by employing the above ECIAF model to examine variation in malnutrition over successive periods in selected South Asian regions. The results are as under.

Table 2 reveals that as the single growth retardation symptom, stunting is the largest among all the other problems of undernutrition over all the periods. Similarly, the greatest number of children is affected by the double burden of stunting and underweight among all the possible combinations. Wasting and overweight problems have been increased from (1991–2002) but reduced later while the co-occurrence of wasting and underweight increased in the same time cohort, reduced between 2002 and 2013 but rose again in 2018. The incidence of a double burden of stunting and overweight has also the same trend.

**Table 2:** Coexisting anthropometric failures according to the different classifications of malnutrition among children in Pakistan

Coexisting Prevalence of Anthropometric Failures among Children in Pakistan						
Groups	Possible categories of anthropometric failures	PDHS(1990–1991) n = 4043	NNS(2001–2002) n = 8895	NNS(2011) n = 27,887	PDHS(2012–2013) n = 3153	PDHS(2017–2018) n = 4236
A	No failure	1443(35.7)	4104(46.1)	12,101(43.4)	1441 (45.7)	2287 (54)
B	Wasting only	90(2.2)	346(3.9)	1176(8.9)	92(2.9)	90(2.1)
C	Wasting & Underweight	131(3.2)	441(5)	1538(10)	92(2.9)	128(4.9)
D	Wasting, Stunting & Underweight	197(4.9)	432(4.9)	1408(5)	156(4.9)	119(2.8)
E	Stunting & Underweight	1047(25.9)	1547(17.4)	4924(22.3)	551(17.5)	647(16.6)
F	Stunting only	889(22)	1445(16.2)	4514(27.2)	559(17.7)	782 (24.5)
G	Stunting & Overweight	124(3.1)	236(2.7)	1125(6.1)	152(4.8)	73(1.7)
H	Overweight Only	59(1.5)	198(2.2)	607(4.8)	64(2.0)	72 (1.7)
Y	Underweight Only	63(1.6)	146(1.64)	494(3.9)	46(1.5)	38 (0.9)
**ECIAF**		**64.3**	**53.9**	**56.6**	**54.2**	**46**

ECIAF aggregate detects more undernourished children as compared to stunting, wasting and underweight separately as it identifies 64.3%, 53.9%, 54.2% and 46% more malnourished proportions in children respectively from 1990 to 2018. The prevalence decreases periodically. However, a major percentage improvement has been observed between the periods of 1990 to 2002 (Table 3).

**Table 3:** Comparison of ECIAF with conventional indices among children in Pakistan (under five)

Conventional indices of anthropometric failures	PDHS(1990–1991) n = 4043	NNS(2001–2002) n = 8895	PDHS(2012–2013) n = 3153	PDHS(2017–2018) n = 4236
Stunting	2257 (55.8)	3624(40.7)	1418 (45)	1621(38.3)
Wasting	418 (10.3)	1251(14.1)	331 (10.5)	335 (7.9)
Underweight	1438 (35.6)	2530(28.4)	845 (26.8)	930 (22)
Overweight	183 (4.5)	474(5.3)	225 (7.1)	145 (3.4)
**ECIAF**	**64.3**	**53.9**	**54.2**	**46**

Table 4 expresses that Child malnutrition first increases and then decreases over successive periods. Malnutrition is highest within the time span of 1996-97 and lowest during 2017-18. Regarding single growth retardation problems, stunting is the highest and for double burden of malnutrition, the coexistence of stunting and underweight is highest among all.

**Table 4:** Coexisting anthropometric failures according to the different classifications of malnutrition among children in Bangladesh

Coexisting Prevalence of Anthropometric Failures among Children of Bangladesh								
Groups	Possible categories of anthropometric failures	BDHS (1990–1991) n = 5351	BDHS (1996–1997) n = 4706	BDHS (2004) n = 6186	BDHS (2007) n = 5535	BDHS (2011) n = 7647	BDHS (2014) n = 7256	BDHS (2017-18) n = 7806
A	No failure	2158 (40.8)	1316 (28)	2532 (40.9)	2465 (44.5)	3568 (46.7)	3694 (50.9)	4641 (59.5)
B	Wasting only	94 (4.1)	113 (7.7)	138 (5.1)	169 (6.3)	228 (5.9)	234 (5.9)	194 (3.9)
C	Wasting & Underweight	208 (7.8)	321 (17)	259 (8.3)	352 (10.9)	429 (9.5)	418 (9.1)	241 (4.6)
D	Wasting, Stunting & Underweight	356 (6.7)	529 (11.2)	469 (7.6)	427 (7.7)	528 (6.9)	405 (5.6)	229 (2.9)
E	Stunting & Underweight	1493 (32.1)	1491 (40.8)	1653 (31.4)	1251 (27.6)	1540 (24.3)	1292 (21.2)	1087 (15.6)
F	Stunting only	827 (27.5)	746 (36.2)	909 (26.4)	625 (20.2)	992 (21.8)	870 (19.1)	1091 (19)
G	Stunting & Overweight	22 (0.7)	57 (2.7)	31 (0.9)	17 (0.5)	50 (1.1)	35 (0.8)	38 (0.6)
H	Overweight Only	20 (0.8)	31 (1.6)	26 (0.8)	43 (1.3)	71 (1.6)	64 (1.7)	114 (2.4)
Y	Underweight Only	146 (6.2)	102 (7)	169 (6.2)	186 (6.9)	241 (6.2)	243 (6.2)	171 (3.5)
**ECIAF**		**59.2**	**72**	**59.1**	**55.5**	**53.3**	**49.1**	**40.5**

ECIAF showed a higher prevalence of undernutrition in comparison to three traditional indicators that is stunting, wasting, overweight and underweight among all time spans. Therefore, it is established that ECIAF is a better indicator of child nutritional status than traditional measures because it determines overall anthropometric failure (Table 5).

**Table 5:** Comparison of ECIAF with conventional indices among children of Bangladesh

Coexisting Prevalence of Anthropometric Failures among Children of Bangladesh							
Conventional indices of anthropometric failures	BDHS (1990–1991) n = 5351	BDHS (1996–1997) n = 4706	BDHS (2004) n = 6186	BDHS (2007) n = 5535	BDHS (2011) n = 7647	BDHS (2014) n = 7256	BDHS (2017-18) n = 7806
Stunting	2698 (50.4)	2823 (60)	3062 (49.5)	2320 (41.9)	3110 (40.7)	2602 (35.9)	2445 (31.3)
Wasting	656 (12.3)	958 (20.4)	866 (14)	948 (17.1)	1184 (15.5)	1057 (14.6)	660 (8.5)
Underweight	2203 (41.2)	2443 (51.9)	2550 (41.2)	2216 (40)	2738 (35.8)	2359 (32.5)	1728 (22.1)
Overweight	44 (0.8)	93 (2)	57 (0.9)	60 (1.1)	122 (1.6)	100 (1.4)	156 (2)
**ECIAF**	**59.2**	**72**	**59.1**	**55.5**	**53.3**	**49.1**	**40.5**

Table 6 shows that child anthropometric failures are highest during 1992-92 and lowest during 2015-16. The stunting prevalence among children is highest within all-time spans and a coexistence of stunting and underweight is highest with respect to the double burden of growth retardation.

**Table 6:** Coexisting anthropometric failures according to the different classifications of malnutrition among Indian children

Coexisting Prevalence of Anthropometric Failures among Indian children					
Groups	Possible categories of anthropometric failures	IDHS (1992–1993) n = 26,755	IDHS (1998–1999) n = 24,855	IDHS (2005-6) n = 43,498	IDHS (2015-16) n = 225,002
A	No failure	8958 (33.5)	9337(37.6)	18,497(42.5)	99,224(44.1)
B	Wasting only	977(9.8)	1001(9.7)	1934(9.3)	13,587(12)
C	Wasting & Underweight	1537(12.8)	1499(12.1)	2771(11.3)	18,063(13.3)
D	Wasting, Stunting & Underweight	2517(9.4)	2088(8.4)	3223(7.4)	14,492(6.4)
E	Stunting & Underweight	7077(33.8)	5580(28.8)	9161(26.5)	39,753(22.9)
F	Stunting only	4346(32.7)	3960(29.8)	6006(24.5)	29,052(20.5)
G	Stunting & Overweight	490 (3.5)	536(3.8)	531(2.1)	3016(2.3)
H	Overweight Only	321(3.5)	338(3.5)	444(1.8)	2508(2.5)
Y	Underweight Only	532(5.4)	516(5.2)	931(4.7)	5307(5.1)
**ECIAF**		**66.5**	**62.4**	**57.5**	**55.8**

Table 7 depicts that measurement of underweight, stunting, wasting and overweight under-estimates the burden of malnourishment. Although conventional indices gives valuable information and must not be disregarded, ECIAF itself is constructed from the aggregation of these indices. Yet the Composite index of Anthropometric failure (ECIAF) better estimates the burden of undernutrition as it reveals additional dimensions of the malnutrition “iceberg”.

**Table 7:** Comparison of ECIAF with conventional indices among children (under five)

Conventional indices of anthropometric failures	IDHS (1992–1993) n = 26,755	IDHS (1998–1999) n = 24,855	IDHS (2005–2006) n = 43,498	IDHS (2015–2016) n = 225,002
Stunting	14,429(53.9)	12,163(48.9)	18,921(43.5)	86,312(38.4)
Wasting	5000(18.7)	4557(18.3)	7900(18.2)	45,915(20.4)
Underweight	11,663(43.6)	9683(39)	16,086(37)	77,615(34.5)
Overweight	843(3.2)	906(3.6)	1003(2.3)	5752(2.6)
**ECIAF**	**66.5**	**62.4**	**57.5**	**55.8**

**Fig. 1:**
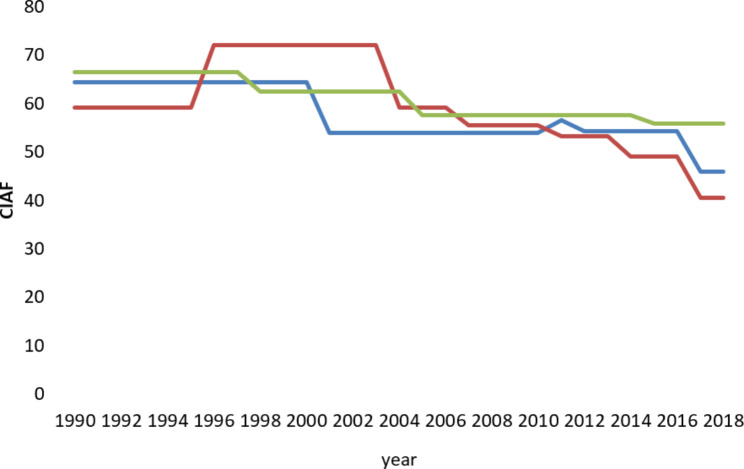
The Blue line represents CIAF figure for Pakistan, the Red line for Bangladesh and the Green line represents India from 1990 to 2018

Figure 1. **Trends of ECIAF over time among children in Pakistan, Bangladesh and India**.

The following graph shows the different time trends in child malnutrition in Pakistan, Bangladesh and India from 1990 to 2018.

## Panel econometric analysis

### Panel unit root test

We cannot apply first- generation panel unit root tests to test the stationarity of the variables as cross-section units are not cross-sectionally independent. We will use the second-generation panel unit root test of Pesaran, (2007) that allows for cross-section dependence [64]. The panel unit root test confirms that all the variables are stationary at first difference.

Table 8 shows the descriptive statistics of all the variables used in the current analysis, their central tendency, maximum and minimum values, variability and distribution.

**Table 8:** Descriptive analysis of the outcome and exposure variables

Variables	Mean	Maximum	Minimum	Std. Dev.	Skewness	Interquartile range	Kurtosis
ECIAF	59.36	72	40.5	7.04	-0.08	10	3.00
Dependency ratio	68.39	89.37	48.95	11.63	0.23	18.20	2.08
Household size	6.91	9.33	4.71	1.54	0.28	3	1.54
Female Literacy rate	42.58	71.18	16.4	13.61	0.12	24	2.16
Prevalence of undernourishment	17.29	59.30	10.8	5.35	5.63	3.50	44.86
Anemia in children	60.74	77.10	40.3	8.66	-0.32	12	2.76
Air pollution	63.84	95.24	51.64	10.29	1.01	14.48	3.56
Unimproved sanitation	23.11	47.85	2.14	14.96	0.05	30	1.59
Political stability	0.622	0.85	0.41	0.12	0.32	0.23	2.22

## Discussion of the FMOLS results

The study analysed pooled weighted FMOLS as proposed by Pedroni (2001) and Kao and Chiang (2000) [65, 66], to allow different long- run variances across the cross section for heterogeneous panels. The test statistics presented in Table 9 showed that all variables are significant and the t value of dependency ratio, household size, government stability, and female literacy rate, Prevalence of undernourishment among the studied population, access to unimproved sanitation, air pollution and anemia in children under five strongly affect child anthropometric failures and performing a significant role in reducing under-five child malnutrition.

**Table 9:** Results of FMOLS

Variables	FMOLS
Dependency ratio	0.457622***(14.59788)
Household size	0.126385***(1.992739)
Female Literacy rate	-0.139380***(-1.221118)
Prevalence of undernourishment	0.107692***(2.650631)
Anemia in children	0.542698***(7.704404)
Air pollution	0.259938***(3.293111)
Unimproved sanitation	0.210710***(3.353972)
Government stability	-4.591377***(-60.82759)
R^2^	0.835740
Adjusted R^2^	0.813239
S.E. of regression	3.005688
Observations	87

t values are given in parentheses

*** (*) shows that the value is statistically significant at 1% (10%) level

The findings in the case of dependency ratio and household size depict positive and significant effects on the prevalence of composite growth retardation problems among children under five and unveil that one unit increase in both the variables positively influences undernourishment by 0.46 and 0.13 units respectively. Yaya et al. (2020), Ghimire et al. (2020) and Ahmad et al. (2020) are also of the view that household structure and family size a risk factors for child malnutrition [56, 67, 68]. Annim et al. (2013) explored the dual effects of household composition and dependency on nutritional outcomes of children under five and found that children of nucleated households with fewer dependents have better health outcomes compared with children in non-nucleated households [69]. Likewise, Fentaw et al. (2013) revealed that households, characterized by more dependency ratio, are more likely to have undernourished children [70].

The literacy rate among women of reproductive age is found to be negative and significant. Kundu et al. (2022), Sk et al. (2021) and Kousar et al. (2020) determined, a lack of maternal education is a common factor in coexisting prevalence of child anthropometric failures among children under five, in South Asia [71–73]. Prevalence of undernourishment within the studied population and anemia in children under five would also increase anthropometric failures among children. In the case of air pollution and unimproved sanitation facilities, our results show that one unit increase in both variables would increase child malnutrition by 0.26 and 0.21 respectively. A study by Gupta and Borkotoky (2016) also confirmed the higher percentage of children having multiple anthropometric failures in households that did not have improved toilet facilities [74]. While few studies have addressed links between air pollution and child health. Bora, (2021), Malley et al. (2021) and Sinharoy et al. (2020) identified that ambient air pollution, slightly increased the risk of anthropometric failures among children under five [43, 75, 76].

The elasticity coefficient of government stability is found to be negative and significant. Thus, there is an inverse relationship between government stability and child malnutrition in South Asian countries and the higher the government stability, the lower would be the prevalence of growth retardation among children and vice versa. Aziz et al. (2021) also explored that the quality of governance is nuanced in declining the rate of undernourishment in South Asia [18].

**Table 10:** Results of Johansen- Fisher Panel Cointegration Test

Johansen- Fisher Panel Cointegration Test				
	Individual Intercept		Individual Intercept and Trend	
	Fisher statistic trace test	Probability	Fisher statistic (Max eigen test)	Probability
None	194.5	0.0000	72.56	0.0000
At most 1	356.2	0.0000	122.6	0.0000
At most 2	167.8	0.0000	90.63	0.0000
At most 3	108.4	0.0000	31.21	0.0000
At most 4	82.41	0.0000	23.60	0.0006
At most 5	65.61	0.0000	21.69	0.0014
At most 6	52.55	0.0000	23.17	0.0007
At most 7	39.76	0.0000	30.16	0.0000
At most 8	20.09	0.0027	20.09	0.0027

H_0_: variables are not cointegrated. All variables are distributed normally, N (0, 1). *** And ** mean significant at 1% and 5% respectively. Fisher’s (1932) test is employed irrespective of an independent variable. Lag intervals for test: 1 1, Asymptotic p-values are estimated by employing χ^2^ distribution tests [77]. Here we see that in the case of none hypothesized cointegrated equation; the probability value is less than 0.05. Therefore we reject our null hypothesis and conclude that the variables have a long- run association. In the same fashion, in at most 1, at most 2 and all the remaining hypothesized conintegrated equations, all the probabilities are less than 0.05. Therefore we reject our null hypothesis and state that the variables under consideration are conintegrated and have long- run associations (Table 10)

Table 11 shows that as the probability of ADF test statistic is less than 0.05, we reject our null hypothesis and conclude that the variables are cointegrated and have a long- run association.

**Table 11:** Results of the KAO Residual Conintegration Test

KAO Residual Conintegration	
ADF Test statistic	-3.292257
Significance	0.0005

Null Hypothesis: No cointegration

Trend assumption: No deterministic trend

Lag length: 1

## Limitations of the study

This study is based on DHS cross-sections that are usually conducted with a huge time gap. Reliable and timely data information on child health status is essential for public health interventions, policy making, monitoring progress and reaching the health- related MDGs. Therefore, lack of information and its influences may allow harmful exposures to go undetected, resulting in missed opportunities to improve prevention, health promotion, and treatment interventions. Secondly, there are various factors causing undernutrition in preschool-aged children like mother’s nutritional status, age at first pregnancy and some paternal characteristics that can’t be not examined due to the data’s unavailability. Thirdly, ECIAF does not determine the impact of micro nutrient deficiencies and clinical correlation is not possible as well.

## Conclusion

We conclude that ECIAF accurately measures the overall burden of undernutrition and overnutrition than conventional indices. Since underestimating the size of hidden vulnerable subgroups might deprive a substantial number of children of getting the advantage of extra supplementation and care they urgently need. In this context, this malnutrition index reflects a wider view of the extent and pattern of malnutrition among children under five by exploring further dimensions of the hidden undernutrition “iceberg”. Thus it has potential implications to be used as a tool for screening systems of malnutrition, monitoring of nutritional interventions and tracking achievement of millennium development goals.

Moreover, all the associated risk factors significantly influence the under-five malnutrition problem in all three countries. The education of women of reproductive age (mothers) is a cornerstone for the betterment of child survival and health. An educated mother, through knowledge and awareness, can better deal with risk factors associated with child malnutrition. Thus improvement in mothers’ education improves the feeding and weaning practices of their children. Household demographics also play an important role in the healthy upbringing of their children. The findings of this research also implied that children less than 5 years of age living in houses with access to unimproved sanitation facilities and air pollution had increased the danger of child malnutrition. Children learn to crawl and walk at this stage of their age, and can experience more exposure to pathogens that are the prime cause of diarrhea from different environmental sources. Similarly, emerging environmental threats including air pollution have also been linked to an increased risk of childhood anthropometric failures. Political stability is also found to be an essential determinant of child health outcomes and growing political stability leads to strengthened social and health programs, that may reduce child malnutrition.

## Recommendations

Unquestionably, the cumulative impact of the several underlying factors escalating the severity of undernutrition and overnutrition problems in South Asian countries is thought-provoking and requires considerable commitment and solemnity by the governments to address this issue. At first ECIAF approach should be adapted to accurately measure and recognize the size of all hidden vulnerable groups. This measurement model can accelerate the reduction in child mortality by expanding preventive and curative interventions that are more effective in addressing the significant causes of undernutrition.

Similar studies should be undertaken among other ethnic preschool children, especially in rural areas, to determine the extent of malnutrition using ECIAF. Such studies would help us to generate new data, that can be used for comparison with the pervasiveness of malnutrition in the regional, national and global context. Better health and nutritional policies can be formulated based on the findings of those investigations.

Although malnutrition can manifest in multiple ways the path to prevention is virtually identical. Adequate maternal nutrition before and during pregnancy and while breastfeeding should be ensured at any cost. Focus on the first crucial thousand days, and promote healthy feeding and weaning practices among the children to combat obesity. Give your child nutritious, diverse and safe foods in early childhood and a healthy environment, including access to basic health, water, hygiene and sanitation services and opportunities for safe physical activity. Improving overall household living conditions and increasing maternal nutritional awareness and knowledge can lead to reduced childhood malnutrition. Besides, an improved status for women should be prioritized.

At the national level, efforts should be made to improve the environment and air pollution conditions may need to be considered an integral part of the programmatic responses by governments and development partners for the prevention of under-five child health status. To combat the occurrence of undernourishment and anemia, the authorities should take steps to improve the quantity and quality of available food as well as food prices should also be controlled and managed at a pace to make food affordable for the commons. Enhance food and nutrition knowledge at the community level by initiating education programs and public awareness campaigns to maintain healthy lifestyles and dietary practices. The health ministry and the government should prioritize and initiate nutrition intervention programs of food fortification with multiple micronutrients and additional supplementation of vitamin A at the national, provincial and district levels with special emphasis on children as the builders of the nation. Last but not least, generating political commitment to ending all forms of malnutrition signifies a key challenge for the global nutrition community. The policies, programs, and resources needed to improve nutrition should be implausibly adopted, effectively implemented, or sustained, without commitment.

## Data Availability

All the data sets and materials used for this research are available on request from the corresponding author DHS data sets: https://www.dhsprogram.com/data/available-datasets.cfm?ctryid=31.

## Abbreviations

CIAF: Composite Index of Anthropometric Failure
FMOLS: Fully Modified Ordinary Least Square
WHO: World Health Organization
ECIAF: Extended Composite Index of Anthropometric Failure
GDP: Gross Domestic Product
UNICEF: United Nations Children’s Fund
HAZ: Mean Height for Age Z Scores
WHZ: Mean Weight for Height Z Scores
WAZ: Mean Weight for Age Z Scores
PDHS: Pakistan Demographic and Health Survey
NNS: National Nutrition Survey
IDHS: Indian Demographic and Health Survey
BDHS: Bangladesh Demographic and Health Survey
WDI: World Development Indicators
WASH: Water, Sanitation and Hygiene
ICRG: International Country Risk Guide
GBD: Global Burden of Disease
IHME: Institute for Health Metrics and Evaluation
GHDx: Global Health Data Exchange
ENA: Emergency Nutrition Assessment
SPSS: Statistical Package for Social Sciences
SD: Standard Deviation
